# Lower expression of genes near microRNA in *C. elegans *germline

**DOI:** 10.1186/1471-2105-7-112

**Published:** 2006-03-06

**Authors:** Hidenori Inaoka, Yutaka Fukuoka, Isaac S Kohane

**Affiliations:** 1 Department of Biosystem Modeling, School of Biomedical Science, Tokyo Medical and Dental University, Tokyo, Japan; 2 Department of Biomedical Information, Institute of Biomaterials and Biomedical Engineering, Tokyo Medical and Dental University, Tokyo, Japan; 3 Informatics Program, Children's Hospital, Harvard Medical School, Boston, MA, USA; 4 Division of Health Sciences and Technology, Harvard University and MIT, Cambridge, MA, USA; 5 Harvard Partners Center for Genetics and Genomics, Boston, MA, USA

## Abstract

**Background:**

MicroRNAs (miRNAs) are recently discovered short non-protein-coding RNA molecules. miRNAs are increasingly implicated in tissue-specific transcriptional control and particularly in development. Because there is mounting evidence for the localized component of transcriptional control, we investigated if there is a distance-dependent effect of miRNA.

**Results:**

We analyzed gene expression levels around the 84 of 113 know miRNAs for which there are nearby gene that were measured in the data in two independent *C. elegans *expression data sets. The expression levels are lower for genes in the vicinity of 59 of 84 (71%) miRNAs as compared to genes far from such miRNAs. Analysis of the genes with lower expression in proximity to the miRNAs reveals increased frequency matching of the 7 nucleotide "seed"s of these miRNAs.

**Conclusion:**

We found decreased messenger RNA (mRNA) abundance, localized within a 10 kb of chromosomal distance of some miRNAs, in *C. elegans *germline. The increased frequency of seed matching near miRNA can explain, in part, the localized effects.

## Background

MicroRNAs (miRNAs) are short (~22 nt-long) non-protein-coding RNAs. In metazoans, miRNA initially thought to be primarily involved in post-transcriptional control [1] have now been shown to have profound and tissue-specific effects on mRNA transcript abundance across significant fractions of the transcriptome [2]. Concurrently it has been demonstrated that miRNAs have a central role in development and organogenesis [3,4]. In the context of the apparent interactions between miRNA and transcriptional control and mounting evidence for the localized component of transcriptional control [5,6], we performed a genome-wide study of *C. elegans *to determine a) if there were localized effects of miRNA on transcription and b) if previously identified "seed matching" between miRNA and their gene targets could explain, in part, the observed decrease in expression around some miRNAs.

## Results and discussion

First, the 113 known miRNAs in *C. elegans *were mapped to the worm genome and genes near each miRNAs were sought. Ninety-six miRNAs were found with at least one gene within 10 kb and 31 miRNA were found within the introns of protein-coding genes. Detailed information about the miRNAs is found in supplemental data. Two experiments of genome-wide expression profiling in *C. elegans *were analyzed. The first dataset by Kim et al. [7] included various growth and developmental conditions and the second investigated the *C. elegans *germline [8]. The number of genes whose positional information was available was 15,281 and 15,859, respectively (detailed information on the datasets and miRNAs is found in Additional files 1, 2, 3). Of the 96 miRNAs with at least one gene in the 10 kb vicinity, there were 12 miRNAs for which no microarray expression measurement was available for those genes in the vicinity (in both datasets). In the Kim data, the expression level of one gene near one of the 84 miRNAs was not correctly measured and thus 83 miRNAs had valid expression data in the 10 kb vicinity.

**Figure 1:**
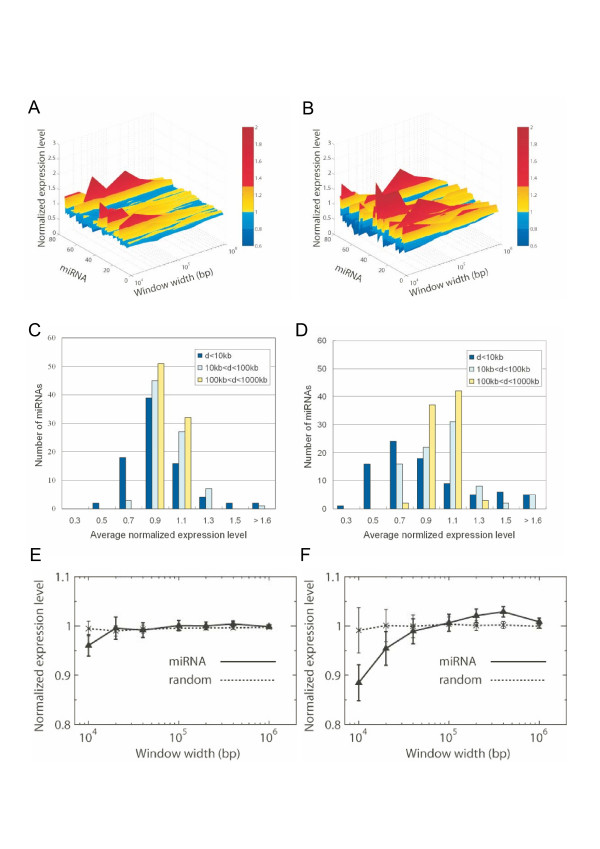
Localized decrease in mRNA levels near miRNAs. A and B, The expression levels around each miRNA are plotted against the window width (A: data by Kim et al. [7] and B: germline data [8]). It should be noted that only 83 and 84 miRNAs are shown in A and B, respectively. This is because no protein-coding genes are found near the other miRNAs. Also miRNAs, which were close to each other, having the same expression values of surrounding genes were grouped and treated as a cluster. The information on the numbering of the axis labeled "miRNA" in Figure 1A and 1B is found in supplemental files. C and D, Distributions of the normalized average expression in the 10 kb window (C: Kim and D: germline data) (see the navy blue bars). In these histograms, distributions of the normalized averages in other distance ranges are also plotted: the light blue and yellow bars represent a distance range between 10 kb and 100 kb and a distance range between 100 kb and 1000 kb, respectively. E and F, The average expression levels over all miRNAs are shown (E: Kim and F: germline data). The normalized expression levels of randomly selected genes are also shown in these graphs.

The normalized average expression levels (see Methods) in the vicinity of the 83–84 miRNAs in *C. elegans *were calculated over several successively wider "windows" of physical distance each centered on the position of a specified miRNA. Figure 1A and 1B show the normalized expression levels for each miRNA in the Kim and germline datasets, respectively. It should be noted that these graphs show only the miRNAs with at least one gene in the narrowest window of 10 kb width. In what follows, we will refer to such a miRNA as "miRNA with gene(s) in the 10 kb window." Because some miRNAs, which were close to each other, were surrounded by the same protein-coding genes, such miRNAs were grouped as a cluster. There were five such clusters in both datasets and each cluster was treated as one entry in Figure 1. Each miRNA or cluster is assigned a number according to its chromosomal position and this number was used for the numbering on the axis labeled "miRNA". Detailed information on the numbering is found in Additional files 4 and 5. These graphs reveal that the normalized expression levels are smaller than 1.0 near the majority of miRNAs in the germline dataset.

In the Kim data, the normalized expression levels near 59 out of the 83 miRNAs with gene(s) in the 10 kb window are smaller than 1.0. In the germline data, the corresponding number is 59 out of the 84 miRNAs with gene(s) in the 10 kb window. These miRNAs are considered to be miRNAs with low-expression genes in their vicinity. Of these miRNAs, 49 are common in both datasets. Of the miRNAs with high-expression genes, 15 are common in the datasets. Distributions of the normalized average expression in the 10 kb window are shown in Figure 1C (Kim) and 1D (germline) (see the navy blue bars). In these histograms, distributions of the normalized averages in other distance ranges are also plotted: the light blue and yellow bars represent a distance range between 10 kb and 100 kb and a distance range between 100 kb and 1000 kb, respectively.

We investigated whether or not the decreased level of gene expression was observable in the average over all the 83–84 miRNAs although we recognize that the miRNAs are hardly the only regulators of gene expression level and that many other factors come into play in determining levels (such as trans-acting transcription factors). Figures 1E and 1F show the averaged expression levels calculated in each sized window over the 83–84 miRNAs. These graphs revealed a marked decrease in transcriptional abundance of those genes in proximity to miRNA, most evident within 10 kb or less. This contrasts with the expression levels relative to 1,130 randomly picked locations in the *C. elegans *genome that revealed no such proximal decrease (see the dashed line in Figures 1E and 1F). In each sized window, the average expression of the genes around miRNA was compared with that of the randomly selected genes. Results of t test indicate that in the germline dataset, the expression of the genes around miRNA is significantly lower (p < 0.05) than that of the randomly selected genes in the 10 kb window. In contrast, in the Kim dataset, the tendency does not reach significance.

**Figure 2:**
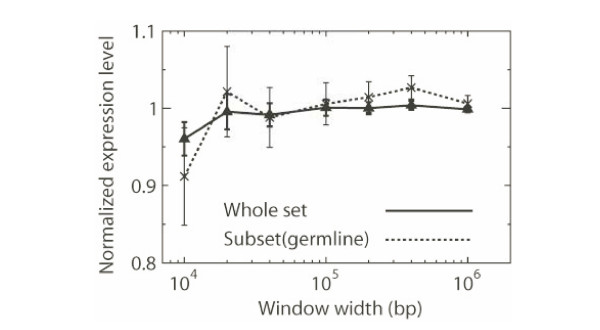
A subset of Kim data, which were obtained from germ cells, showed a stronger effect.

When comparing the results from the two datasets (Figures 1E and 1F), the germline data show a clearer decrease in proximity to miRNA. This may be explained by the following facts. First, the dataset by Kim *et al*. includes a wide variety of samples from several growth conditions, developmental stages and varieties of mutants [7]. As mentioned earlier, miRNAs appear to play an important role in development, organogenesis and gametogenesis [3,4,9]. Although the Kim dataset includes some developmental datasets, the development-specific effects of miRNA may be diluted by the variety of other conditions included. In contrast, the germline dataset contains samples from mutants with defects in germline proliferation or gamete production [8] so the stage-specific miRNA effect is less likely to be muddied. In fact, a subset of Kim data, which were obtained from germ cells, showed a stronger effect (Figure 2).

There is a possibility that the genes in proximity to miRNA are not real genes and thus the decreased expression level near miRNA can be explained by low expression of such genes. We examined the possibility in the following way. The expression levels of the 139 genes in the narrowest window in the germline data were investigated in all experimental conditions to see if there were any times when those genes did not have low values (Figure 1 shows only the normalized average values). This analysis revealed that the 139 genes were highly expressed (the normalized level exceeded 2) in various experiments (detailed information is found in Additional file 6) and that it was just their average that was low. This suggests that those genes with low expression near miRNA are real, functioning genes.

The relationship between miRNAs and their putative gene targets were analyzed to see if these accounted for any of the observed decrease in expression. The numbers of matching sites in the genes to bases 2–8 of the mature forms of the miRNAs, which are termed the "seeds" of the miRNA [10], were counted. Seed matching between miRNA and their target genes have previously been found to have predictive value on transcript abundance [10,11]. The distribution of such seed matching was investigated to determine if it could account for mRNA transcript abundance and for the observed local decrease of expression around miRNA.

First, to examine whether there was any relationship between seed matching and expression levels (regardless of distance), we compared two extreme groups. Of the genes in the 1000 kb windows, we selected the 5 % fraction with the lowest expression levels (Lowest5%) and the same number of genes from the highest expression levels (Highest5%). Then, the number of the seed matches between those genes and miRNAs were counted. To reduce the computational time, we analyzed only pairs which are on the same chromosome. The Lowest5% group had 337 seed matches whereas 226 matches were found in the Highest5% group. This revealed that the number of the seed matches obtained from the Lowest5% genes is greater than that from Highest5%, supporting a relationship between the number of seed matches and expression levels.

**Figure 3:**
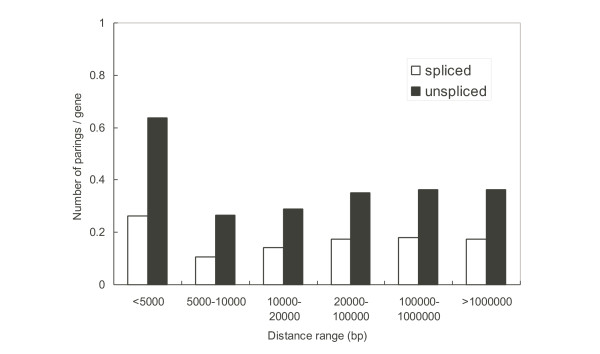
The number of seed pairings around genes in different distance ranges. Both spliced and unspliced mRNA forms of protein-coding genes were investigated.

Further, we counted the number of the seed matches (i.e. potential targets) around the protein-coding genes selected from the following six distance ranges: i) < 5 kb, ii) 5 – 10 kb, iii) 10 – 20 kb, iv) 20 – 100 kb, v) 100 – 1000 kb and vi) > 1000 kb. Seed matches with the messenger of each protein coding gene (both spliced and unspliced) were identified. The number of the seed matches per gene is shown in Figure 3. It was much larger in the unspliced mRNA in the smallest distance range (< 5 kb). Table 1 summarizes the relationship between the expression level and the number of seed matches. In this table, the numbers of seed matches in the upstream and downstream regions are also given. The expression levels in this table represent the normalized average expression levels of the genes with seed matches in the smallest range. Genes with seed matches in their message forms had lower expression levels than those with seed matches in other parts of the gene. Because the 3' untranscribed region (UTR) was included in the unspliced mRNA in this analysis, the results are consistent with the identification of validated miRNA targets found in the 3' UTR of genes [10,11] although other sites for targeting have been identified. Each dataset was divided into two groups according to whether the normalized expression level in the 10 kb window exceeded 1.0 (high) or not (low). The analysis of seed matches was repeated for those subsets. The results (Table 2) provide further evidence for a relationship between expression level and the number of seed matches. These findings of increased seed matching of proximal genes that have low expression levels and the over-representation of seeds in the 3' end of putative gene targets provide a potential mechanism for the observed localized decrease in expression in the vicinity of miRNA. Neither 7-bp random sequences taken from non-protein-coding regions nor computationally generated random sequences could reproduce these results (see supplemental information in Additional files 7 and 8). These results indicate that the results in Figure 3 reflect true biological phenomena and further suggest that the increased number of seed matches near miRNA could explain, in part, the observed localized decrease.

We investigated the expression levels of the genes near the miRNAs exist in introns of other protein-coding genes and the expression levels of the host genes. However, there appeared to be no clear relationship (see Additional file 2 for the details). This suggests that the localized decrease is not due to the miRNAs in introns.

We identified Gene Ontology (GO) [12] categories with an overrepresented number of genes near miRNA (Table 3). Of the genes in WormBase, 11,420 had GO molecular function annotations while 118 genes (regardless of the availability of expression data) near miRNA had annotations in molecular function. The number of the GO molecular function categories including two or more genes out of the 118 was 21. 16,363 and 105 GO biological process annotations were found in the whole dataset and the genes near miRNA, respectively. The number of the GO biological process categories including two or more genes out of the 105 was 20. The χ^2 ^test revealed that one category included a greater number of genes than expected by chance (p < 0.0024, a corrected value according to the Bonferroni method). The category was GO7165 (signal transduction) in biological process. Two GO biological process categories with overrepresented numbers of genes were obtained when this analysis was repeated using only the genes with seed match near the 49 common miRNAs with low-expression genes (GO7165) and near the 15 common miRNAs with high-expression genes (GO6464) (Table 3). We also used the KEGG (Kyoto Encyclopedia of Genes and Genomes [13]) pathways which includes an overrepresented number of genes near miRNA. Of the genes in WormBase, 1,642 were involved in at least one pathway while 28 of the genes (regardless of the availability of expression data) near miRNA were found in the pathways. The number of the pathways including two or more genes out of the 28 genes was 6. The χ^2 ^test indicated that no pathway included a greater number of genes than expected by chance (p < 0.0083, a corrected value according to the Bonferroni method). When this analysis was repeated using only the genes with seed match near the common miRNAs with low- or high-expression genes, no pathway was found to be enriched. Although the biological importance of these results is currently obscure, these may provide insights into target genes and mechanisms of transcriptional control with miRNAs.

Without attempting to draw broader conclusions about these mechanisms we performed a similar analysis on the murine homologues of those miRNA in *C. elegans *that showed the most decreased localized abundance in the above studies. We found similar phenomena in mice. Expression levels of genes around 12 murine miRNAs homologous to cel-mir-1 and cel-mir-let-7 were lower than the overall average level in three mouse datasets [14,15] (Table 4).

## Conclusion

In this paper, we performed a genome-wide study of *C. elegans *based on two independent studies. We found that there is a decrease of messenger RNA (mRNA) abundance, localized within a 10 kb of chromosomal distance of a majority of miRNAs, in *C. elegans *germline. The precise nature of the miRNA effect on transcription remains unclear even though the evidence of such effects is becoming more evident [2,16]. The localized decrease in expression of genes near 59 miRNAs in *C. elegans *we have found in two experiments may be in part explained by the increased seed matches with proximal target genes but the mechanism of the effect of expression levels remains to be investigated.

## Methods

### Sequences, chromosomal positions and expression data

The gene expression data were retrieved from Stuart Kim Lab [17] and the Gene Expression Omnibus (GEO) at the National Center for Biotechnology Information [18] (Accession Numbers GSE715~GSE737). The first dataset contains 19,641 probes from 533 experiments including several growth conditions, developmental stages and varieties of mutants [7]. A subset of this, in which data were obtained from germ cells, was also analyzed (The subset is available at GEO, Accession Number GDS6). The other set includes 17,996 probes from 76 measurements on the *C. elegans *germline [8]. All information on the sequence and location (chromosome, position and strand) of the 113 miRNAs and the genes in the datasets were obtained from WormBase [19] (as of May '05, Release WS144).

### Effects of the distance between gene and miRNA on expression level

In each of the datasets, the average over all genes in all experiments was calculated. Then the expression levels were normalized using the over all average. A window centered at a miRNA was set, and the average of the normalized expression levels was calculated in the window. When averaging the expression levels, the absolute values of normalized expression levels were used. Seven different window widths were used: 10 kb, 20 kb, 40 kb, 100 kb, 200 kb, 400 kb and 1000 kb. The normalized average expression levels were plotted against the distance from the miRNA at the center of the window. This procedure was repeated using randomly picked positions. We used 1,130 random positions.

### Seed matching between miRNA and presumptive target gene

Although the seed matching rule is not universal in *C. elegans*, the number of seed matches could be a simple, good measure for rough estimation of how many target genes are involved in a specific group. We compared the number of matches in genes with low expression levels to that from highly expressed genes to investigate whether the number of seed matches has any effect on the expression level. Of the 8,447 genes in the 1000 kb windows in the germline data, we selected the 5 % fraction (422 genes) having the lowest expression levels (Lowest5%) and the same number of genes from the highest expression levels (Highest5%) as two extreme groups. Then using BLAST with default settings except the word size option (set at 7), we counted the number of matching sites in the genes to bases 2–8 of the miRNAs on the same chromosome and compared the numbers from both groups.

A similar analysis was performed for the genes in the following distance (from a miRNA) ranges: i) < 5 kb, ii) 5 – 10 kb, iii) 10 – 20 kb, iv) 20 – 100 kb, v) 100 – 1000 kb and vi) > 1000 kb. In this analysis, seed matching sites were sought between a miRNA and the genes in the above ranges from the miRNA. In addition to the mRNA form (both spliced and unspliced) the sequences of 2,500 bp in the upstream and downstream regions were used to compare the results with those from mRNA. It should be noted that in WormBase, the unspliced mRNA form includes the UTR regions. This analysis was repeated using random 7 base seeds to see whether or not the results can be reproduced with the random seeds. In the analysis using the random seeds, 113 seeds were generated according to the following methods: in the first method, 7-bases long sequences were taken randomly from non-protein-coding regions and in the other method, the seeds were generated with a computer. In the latter, each base had the equal probability of occurring at any position although in the worm genome, bases A and T have higher probabilities of occurring than G and C (see Supplemental Table A).

### Gene Ontology categories and KEGG pathways

The Gene Ontology (GO) categories [12] of the genes in the 10 kb windows were analyzed to investigate whether there were any categories which included an overrepresented number of genes near the miRNAs. The GO is built in a hierarchical manner, with a parent-child relationship between categories. Because some categories at lower levels in the hierarchy were too specific for our purpose, we used a set of broader, high-level GO parents known as GO Slim terms (detailed information is found at [20]) in this analysis. First, we performed this analysis using all the genes near the miRNAs with gene(s) in the vicinity regardless of the availability of expression data. Then, the procedure was repeated using only those genes with seed matches in their unspliced mRNA forms near miRNAs with low expression genes in their vicinity. The χ^2 ^score was employed as a measure of statistical significance [21]. It should be noted that GO categories including only one out of the genes near the miRNAs with gene(s) in their vicinity were excluded from the analysis because such a result easily happened by chance. The level of significance was corrected according to the Bonferroni method because the χ^2 ^test was repeated several times. That is, the significance level of 0.05 was divided by the number of GO categories each of which included two or more genes out of the genes near the miRNAs with gene(s) in their vicinity. The KEGG pathways [13] including the genes in the 10 kb windows were also investigated in the same way.

## Authors' contributions

HI developed all software and performed the bioinformatical analysis. YF conceived the methodology, performed the statistical tests and drafted the manuscript. ISK suggested the investigation and guided the study. All authors read and approved the final manuscript.

## Supplementary Material

Additional File 1 an Excel book file, the datasets information. This file provides the detailed information on the probes on the microarrays and expression levels of the genes in the Kim and germline datasets. Click here for file

Additional File 2 an Excel book file, the information on the worm miRNAs. This file provides detailed information on the 113 worm miRNAs. It includes the number of genes around each miRNA and the number of genes available in each dataset. The miRNAs in introns are listed in this file. Click here for file

Additional File 3 an Excel book file, the genes near miRNAs. This provides the list of genes near miRNAs in each dataset. Click here for file

Additional File 4 a text file, the numbering information in Figure 1A. This file provides the numbering information about the axis labeled miRNA in Figure 1A. Click here for file

Additional File 5 a text file, the numbering information in Figure 1B. This file provides the numbering information about the axis labeled miRNA in Figure 1B. Click here for file

Additional File 6 an Excel book file, the expression levels of the genes around miRNAs. This file includes expression levels of genes around miRNAs in each experiment. Click here for file

Additional File 7 a PDF file, the results using random seeds. This file includes a supplemental figure, which shows the results of seed matching analysis using random seeds. Click here for file

Additional File 8 a PDF file, the base usage in the worm genome. This file includes a table providing the information about the probabilities of occurring for the four bases in the worm genome. Click here for file

## Figures and Tables

**Table 1:** Relationship between the expression level and the number of seed pairings in the distance range of 5 kb (the whole dataset).

	Normalized expression level	
	
	Kim data	germline data
splieced mRNA	0.88 ± 0.03 (19)	0.78 ± 0.06 (19)
unspliced mRNA	0.90 ± 0.02 (36)	0.77 ± 0.04 (38)
upstream	0.95 ± 0.02 (53)	0.84 ± 0.05 (53)
downstream	0.92 ± 0.02 (59)	0.85 ± 0.0 4 (61)

The normalized expression level is represented in the form of average ± standard error. Each number in the parentheses denotes the number of genes with a seed pairing. It should be noted that some genes have multiple seed pairings in its mRNA form and upstream or/and downstream regions.

**Table 2:** Relationship between the expression level and the number of seed pairings in the distance range of 5 kb. Each dataset was divided into two groups according to the normalized average of the 10 kb window.

	Normalized expression level			
	
	Kim data		germline data	
	
	low	high	low	high
splieced mRNA	0.87 ± 0.14 (17)	0.93 ± 0.09 (2)	0.67 ± 0.20 (19)	1.12 ± 0.15 (3)
unspliced mRNA	0.85 ± 0.11 (29)	1.02 ± 0.14 (7)	0.70 ± 0.20 (32)	1.09 ± 0.16 (7)

The normalized expression level is represented in the form of average ± standard error. Each number in the parentheses denotes the number of genes with a seed pairing. It should be noted that some genes have multiple seed pairings in its mRNA form and upstream or/and downstream regions.

**Table 3:** Gene Ontology categories found significantly enriched.

GO category	all genes	genes near miRNA (p)	seed parings (unspliced) & low expression (p)	seed parings (unspliced) & high expression (p)
Biological Process				
Total	16,363	105	20	2
GO7165 (signal transduction)	620	11 (0.002)	5 (0.001)	0 (NA)
GO6464 (protein modification)	706	9 (0.039)	1 (0.586)	2 (0.002)

**Table 4:** Expression levels of genes near miRNAs conserved between worm and mice.

	Expression level	
	
	window = 200 kb	window = 1000 kb
Lung development [14]	0.65	1.03
Cerebellar development [14]	0.52	1.13
Broad transcriptome [15]	0.85	0.94
