# Supplementary material for: Lower expression of genes near microRNA in C. elegans germline
Source: BMC Bioinformatics. 2006 Mar 6;7:112. doi: 10.1186/1471-2105-7-112 (PMC1420334; doi:10.1186/1471-2105-7-112)
Supplement: Additional File 7 — a PDF file, the results using random seeds. This file includes a supplemental figure, which shows the results of seed matching analysis using random seeds. [file 1471-2105-7-112-S7.pdf]

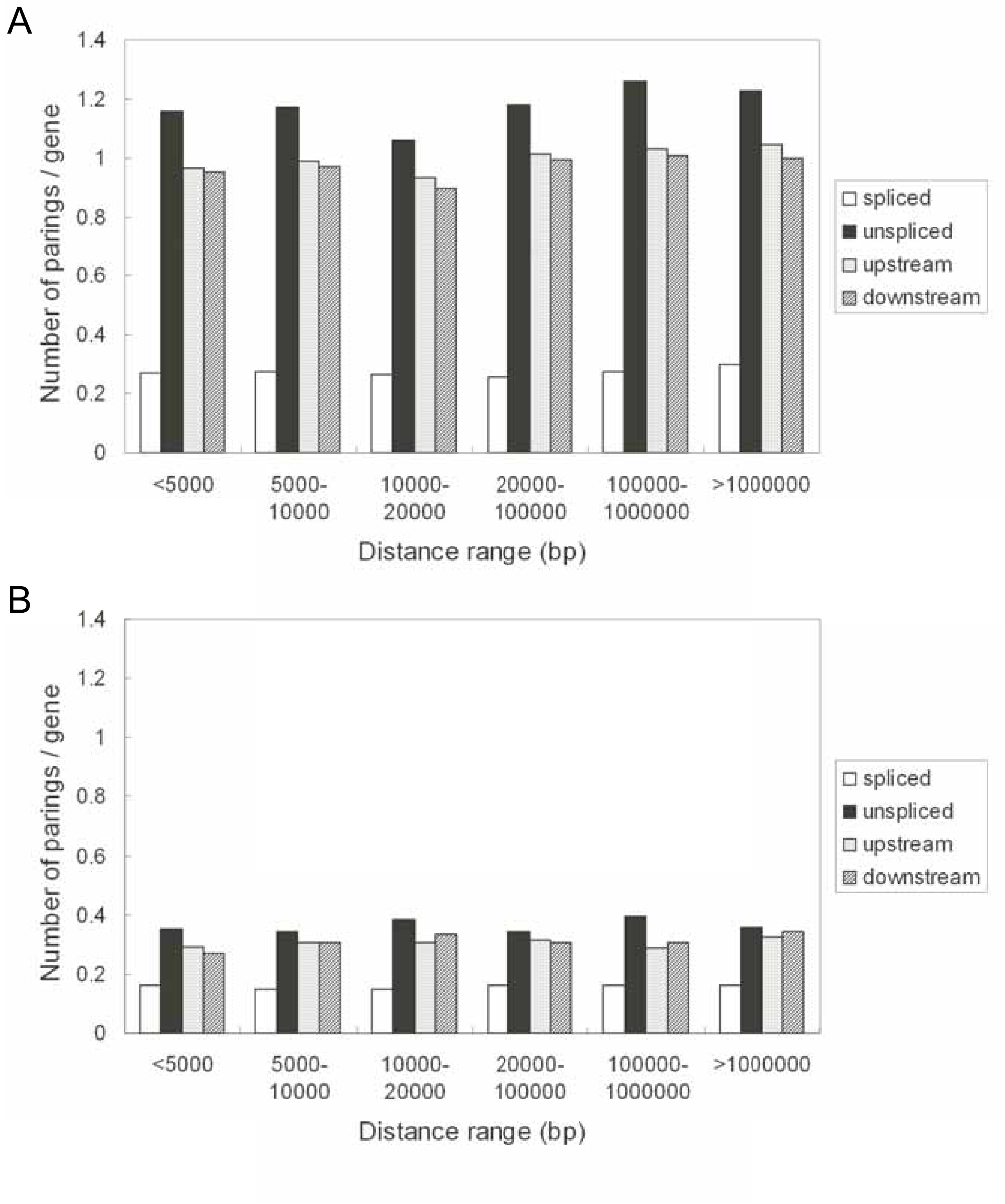

Supplemental Figure A The number of matching sites investigated using 7 bp random seeds.
A, The random seeds were generated by taking 7 bp sequence from non-protein-coding regions.
B, The random seeds were generated computationally. In the random seeds of this type, each base had the equal probability of occurring at any position. Neither type of the seeds could reproduce the results with the true seeds. The discrepancy in A and B is explained by the fact that the base usage in the worm genome (mostly non-protein-coding region) is biased toward bases A and T. These bases have probabilities almost twice as high as those of G and C (see Supplemental Table A). In this case, a 7-bp random sequence has a perfect match in approximately every 2,187 bp (=37 bp) by chance only.
