# Supplementary material for: Lower expression of genes near microRNA in C. elegans germline
Source: BMC Bioinformatics. 2006 Mar 6;7:112. doi: 10.1186/1471-2105-7-112 (PMC1420334; doi:10.1186/1471-2105-7-112)
Supplement: Additional File 8 — a PDF file, the base usage in the worm genome. This file includes a table providing the information about the probabilities of occurring for the four bases in the worm genome. [file 1471-2105-7-112-S8.pdf]

**Supplemental Table A** The probabilities of occurring for the four bases in the worm genome.

| | | A | C | G | T |
|---|---|---|---|---|---|
| whole genome | | 0.323 | 0.177 | 0.177 | 0.323 |
| coding region | | 0.306 | 0.211 | 0.219 | 0.264 |
| miRNA | mature | 0.251 | 0.199 | 0.252 | 0.299 |
| | seed | 0.289 | 0.214 | 0.270 | 0.226 |
